# The Use of Laser Microdissection in the Identification of Suitable Reference Genes for Normalization of Quantitative Real-Time PCR in Human FFPE Epithelial Ovarian Tissue Samples

**DOI:** 10.1371/journal.pone.0095974

**Published:** 2014-04-28

**Authors:** Jing Cai, Tao Li, Bangxing Huang, Henghui Cheng, Hui Ding, Weihong Dong, Man Xiao, Ling Liu, Zehua Wang

**Affiliations:** 1 Department of Obstetrics and Gynecology, Union Hospital, Tongji Medical College, Huazhong University of Science and Technology, Wuhan, China; 2 Department of Pathology, Union Hospital, Tongji Medical College, Huazhong University of Science and Technology, Wuhan, China; 3 Department of Pathology, Tongji Hospital, Tongji Medical College, Huazhong University of Science and Technology, Wuhan, China; National Cancer Institute, National Institutes of Health, United Statesof America

## Abstract

Quantitative real-time PCR (qPCR) is a powerful and reproducible method of gene expression analysis in which expression levels are quantified by normalization against reference genes. Therefore, to investigate the potential biomarkers and therapeutic targets for epithelial ovarian cancer by qPCR, it is critical to identify stable reference genes. In this study, twelve housekeeping genes (ACTB, GAPDH, 18S rRNA, GUSB, PPIA, PBGD, PUM1, TBP, HRPT1, RPLP0, RPL13A, and B2M) were analyzed in 50 ovarian samples from normal, benign, borderline, and malignant tissues. For reliable results, laser microdissection (LMD), an effective technique used to prepare homogeneous starting material, was utilized to precisely excise target tissues or cells. One-way analysis of variance (ANOVA) and nonparametric (Kruskal-Wallis) tests were used to compare the expression differences. NormFinder and geNorm software were employed to further validate the suitability and stability of the candidate genes. Results showed that epithelial cells occupied a small percentage of the normal ovary indeed. The expression of ACTB, PPIA, RPL13A, RPLP0, and TBP were stable independent of the disease progression. In addition, NormFinder and geNorm identified the most stable combination (ACTB, PPIA, RPLP0, and TBP) and the relatively unstable reference gene GAPDH from the twelve commonly used housekeeping genes. Our results highlight the use of homogeneous ovarian tissues and multiple-reference normalization strategy, e.g. the combination of ACTB, PPIA, RPLP0, and TBP, for qPCR in epithelial ovarian tissues, whereas GAPDH, the most commonly used reference gene, is not recommended, especially as a single reference gene.

## Introduction

Despite advances in the understanding of potential biomarkers and therapeutic targets, effective screening techniques and therapies of ovarian cancer remain a challenging task, partly due to tissue heterogeneity. Numerous studies have analyzed genetic alterations or gene expression profiles in ovarian cancer by making the comparison between cancerous tissue and normal ovarian tissue. In a normal ovary, epithelial cells, from which the carcinomas primarily originate, only account for a very small percentage of the ovarian cell population, while stromal cells occupy the overwhelming majority [1]. Thus, it is unreliable to analyze gene expression based on RNA or DNA preparations from whole ovarian tissue [2]. To ensure the accuracy and reliability of results, the procurement of homogeneous cells is immensely important.

Laser microdissection (LMD) is a tool that facilitates the microscopic isolation of objective regions without contamination or unwanted tissue components [3], [4]. The dissected tissues, as homogeneous starting materials, can be used for a variety of analyses, such as transcriptomic and proteomic studies [5]. During this process, the use of Rnase-free reagents and cresyl violet staining are recommended to minimize RNA degradation and maintain tissue morphology, respectively [6], [7]. Although fresh cryosections are preferred for nucleic acids extraction using LMD, fresh sections are usually scarce in basic and clinical research. Formalin-fixed, paraffin-embedded tissues (FFPE), which represent an alternative for nucleic acids extraction and downstream experiments, such as quantitative real-time PCR (qPCR), are more widely available in clinical laboratories.

To date, innumerable papers have embraced qPCR as an indispensable method to quantify gene expression at the transcription level because of its high sensitivity, reproducibility, and throughput [8]–[10]. In this approach, when comparing gene expression profiles, accurate normalization is a prerequisite for reliable results. Therefore, the identification of suitable reference genes is crucial in qPCR assays. An ideal reference gene or housekeeping gene (HKG) should be stably expressed in all specimens regardless of tissue type, pathological stage and/or experimental treatment [11], [12]. Regrettably, the commonly used reference genes have shown variability in expression in different tissues and cells [13]–[16].

Thus far, two reports have been published that explored the identification of reference genes for the normalization of qPCR results in ovarian tissues. After examining 20 normal ovarian tissue specimens and 20 serous ovarian cancer specimens, Li *et al.* recommended a combination of PPIA and GUSB genes as a reliable normalization strategy [17]. Fu *et al.* recommended a combination of RPLP0 and RPL4 afer investigating 52 normal, benign, and malignant ovarian tissues samples [18]. However, both studies did not account for tissue heterogeneity and their results were inconsistent.

To understand the current use of reference genes, we performed a PubMed search using the MeSH terms “real-time PCR” and “ovarian cancer” and obtained 128 available articles published from January 1st, 2010 to March 10th, 2013; among the articles, 25 various reference genes were used without any preliminary evaluation of suitability. Although studies have recommended the combination use of reference genes, only 17 of the 128 (13.3%) studies used two or more reference genes for data normalization. In addition, within the other 111 (86.7%) studies that applied a single reference gene, GAPDH was the most frequently used (52, 40.6%), followed by ACTB (33, 25.8%), 18S rRNA (5, 3.9%), and B2M (3, 2.3%). Other genes, such as TBP, PBGD, RPLP0, PUM1 and GUSB, were only used twice (1.6%) or once (0.78%) among the studies that applied a single reference gene.

In summary, the use of reference genes for qPCR is inconsistent, and standardization is urgent, especially for the evaluation of gene expression in tissues with high heterogeneity, such as ovarian tumors. In this study, we collected epithelial components from FFPE ovarian tissues with varying degrees of neoplasia via LMD to identify universal reference genes for qPCR in ovarian epithelial tumor studies.

## Materials and Methods

### Ethics Statement Results

The present study was approved by the ethical committee of Union Hospital, Tongji Medical College, Huazhong University of Science and Technology, China. All patients or their next of kin have provided written informed consent for the collection of samples and subsequent researches.

### Patients and samples

Between January 2011 and July 2013, we collected a total of 50 tissue samples comprising eight normal ovarian tissues (mean age 41.5 years, age range 28 to 63 years), ten benign neoplasms (three serous neoplasms and seven mucinous neoplasms; mean age 35.3 years, age range 14 to 57 years), seven borderline neoplasms (four serous neoplasms and three mucinous neoplasms; mean age 35.57 years, age range 24 to 60 years), 25 malignant epithelial tumors (12 serous adenocarcinomas, three mucinous adenocarcinomas, six endometrioid carcinomas, and four clear cell tumors; mean age 50.2 years, age range 22 to 64 years). The normal tissue samples were excised from patients receiving an adnexectomy due to adenomyosis or myoma or excised from patients receiving a wedge biopsy of the ovaries. The tumor tissues were obtained during surgery, and cases with preoperative treatment were excluded. All specimens used in this study were pathologically verified.

### LMD

Serial sections (n = 3–20, 8 µm) were cut from each FFPE sample and stored at 4°C until use. A 4-µm thick section was also cut for H&E staining. Immediately before LMD, the sections were deparaffinized, stained with 1% cresyl violet (Sigma-Aldrich, St. Louis, Missouri, USA) for 1 minute, dehydrated through 75%, 95%, and 100% alcohol grades for 30 seconds each, and finally immersed in xylene for 3 minutes and air-dried for 1 minute. The microdissection was performed using the Arcturus^XT^ LCM instrument (Applied Biosystems-Life Technologies, Carlsbad, CA, USA) following the manufacturer's protocol. An AutoScan™ analysis software module was implemented when using the ArcturusXT LCM instrument, which allowed the user to visually inspect the regions of interest. Approximately 5000 cells were captured per specimen and subsequently used for the following studies.

### RNA isolation and cDNA synthesis

Total RNA from homogenized tissues was isolated using the RNeasy FFPE kit (Qiagen, Hilden, Germany) according to the manufacturer's protocol. The quality and integrity of the total RNA were measured using the Nanodrop 2000 (Thermo Scientific, Wilmington, DE, USA) and by electrophoresis on a 1% agarose gel. The total RNA (100 ng) was reverse-transcribed into cDNA using a PrimeScript RT reagent kit (TaKaRa Biotechnology, Shiga, Japan) with random hexamer primers and oligo(dT). All reactions were carried out at 37°C for 15 min followed by 85°C for 5 s to inactivate the enzymes.The cDNA was stored at −20°C until use.

### Quantitative real-time PCR

Reference genes (n = 12) frequently used in the last three years in ovarian cancer research were selected to identify the most stable gene for qPCR normalization. Information regarding the 12 candidate genes is provided in Table 1. Primers were generated from published papers or designed using Primer 5 software, and the specificity of each primer was confirmed by primer-BLAST searches. qPCR was performed on an Applied Biosystems StepOnePlus Real-time PCR system (Applied Biosystems, Foster City, CA, USA) using SYBR Green Real-time PCR Master Mix (Toyobo, Osaka, Japan) according to the manufacturer's recommendations. Negative controls (no template in PCR) were performed for each gene, and a melting curve was constructed for each primer to confirm product specificity.

**Table 1:** Candidate reference genes evaluated in this study.

Gene symbol	Gene name	Accession number	Function
GAPDH	Glyceraldehyde-3-phosphate dehydrogenase	NM-001256799.1	gluconeogenesis and Glycolysis
ACTB	Actin, beta	NM-001101.3	Cytoskeletal structural protein
18S	18S ribosomal RNA	NR-003286	Ribosomal RNA
B2M	Beta-2-microglobulin	NM-004048.2	Cytoskeletal protein in cell locomotion
GUSB	Glucuronidase, beta	NM-000181.3	Lysosomal exoglycosidase
PBGD	Porphobilinogen deaminase	NM-001258209.1	Heme biosynthetic pathway
PUM1	Pumilio homolog 1 (Drosophila)	NM-014676.2	RNA binding
PPIA	Peptidylprolyl isomerase A	NM-021130.3	Cyclosporin binding protein
TBP	TATA box binding protein	NM-001172085.1	Transcription by RNA polymerases
HRPT1	Hypoxanthine phosphoribosyltransferase 1	NM-000194.2	Metabolic salvage of purines
RPLP0	Ribosomal protein, large, P0	NM-001002.3	Ribosomal protein
RPL13A	Ribosomal protein L13a	NM-001270491.1	Ribosomal protein

Standard curves of a five-fold dilution series were performed for all candidate genes, and the amplification efficiency and correlation coefficient (R^2^) were generated based on the slope of each standard curve. The equation used to calculate the efficiency was as follows: E% =  [10^(−1/slope)^–1]%. The delta-C_t_ method was used to calculate the relative quantification (Q) of the amplification of the candidate genes according to the following formula: Q = E^−(sampleCt–minCt)^, where minC_t_ =  the lowest C_t_ value observed in a group analyzed for a given primer.

### Stability analysis

Statistical analysis was carried out with SPSS 13.0 statistic software (SPSS, Chicago, IL, USA). Differences were appropriately analyzed by one-way analysis of variance (ANOVA) or a nonparametric (Kruskal-Wallis) test. P<0.05 was considered statistically significant. For the stability analysis of the candidate reference genes, two freely available software programs were applied: NormFinder (http://www.mdl.dk/publicationsnormfinder.htm) and geNorm version 3.5 (http://medgen.ugent.be/~jvdesomp/genorm/). NormFinder, a Microsoft Excel add-in, focuses on calculating a stability value based on intra- and inter-group expression variations for candidate reference genes. A low value represents a low combined variation and, thereby, reveals high expression stability [19]. GeNorm, another Microsoft Excel application, provides a gene stability measure (*M*) and ranks the tested genes by stepwise exclusion of the gene with the highest *M* value. In addition, geNorm calculates the pairwise variations, V_n_/V_n +1_, between each combination of sequential normalization factors (NF), which allows for the identification of the optimal number of reference genes for accurate normalization [16].

## Results

### LMD, quality control, and amplification efficiency

**Figure 1:**
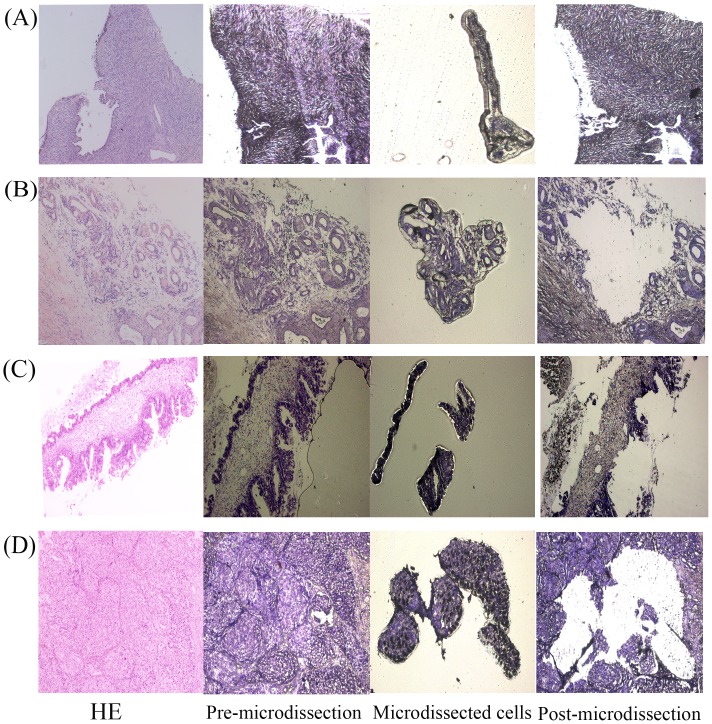
LMD mediated isolation of epithelial cells. (A–D) Ovarian tissue specimens, ordered by normal, benign, borderline, and malignant pathological categories, were used for target cells isolation. Panels from left to right are as follows: sections stained with H&E prior to LMD; sections stained with cresyl violet before LMD; cells captured on the collecting caps; sections after LMD (×100).

Using LMD, approximately 5000 epithelial cells were isolated from each of the normal, benign, borderline, and malignant ovarian tissues (Fig. 1). Results showed that epithelial cells only occupied a small percentage of the whole ovarian tissues, especially the normal ovary. To guarantee the reliability of the results, only high quality RNA samples were included in the subsequent qPCR reactions. The purity and integrity of the RNA samples were characterized by the A260/280 ratio, which ranged from 1.8 to 2.06 (1.92±0.074) and the 28S/18S ratio (>1.7) on a 1% agarose gel. The amplification efficiencies and correlation coefficients (R^2^) of the12 HKGs ranged from 95.1 to 105% and from 0.997 to 1, respectively (Table 2).

**Table 2:** Primer related information.

Gene symbol	Primer sequences (forward/reverse)	Annealing temperature(°C)	PCR efficiency (%)	R^2^	Amplicon length(bp)
GAPDH	F:TGAACGGGAAGCTCACTGG R:TCCACCACCCTGTTGCTGTA	60	95.1	0.999	307
ACTB	F:GCCAACACAGTGCTGTCTGG R:GCTCAGGAGGAGCAATGATCTTG	60	98.7	1	121
18S	F:CGGCTACCACATCCAAGGAA R:GCTGGAATTACCGCGGCT	64	99.5	1	186
B2M	F:CGCTACTCTCTCTTTCTGGC R:AGACACATAGCAATTCAGGAAT	56	105	0.997	113
GUSB	F:AGCCAGTTCCTCATCAATGG R:GGTAGTGGCTGGTACGGAAA	56	105	0.999	160
PBGD	F:AGTGTGGTGGGAACCAGC R:CAGGATGATGGCACTGAACTC	56	103	0.999	144
PUM1	F:CAGGCTGCCTACCAACTCAT R:GTTCCCGAACCATCTCATTC	60	96.8	0.999	211
PPIA	F:GTGGTGTTTGGCAAAGTGAA R:TCGAGTTGTCCACAGTCAGC	60	103	0.999	116
TBP	F:TGCACAGGAGCCAAGAGTGAA R:CACATCACAGCTCCCCACCA	60	95.9	0.999	132
HRPT1	F:TGACACTGGCAAAACAATGCA R:GGTCCTTTTCACCAGCAAGCT	60	102	0.999	94
RPLP0	F:TTAAACCCTGCGTGGCAATCC R:CCACATTCCCCCGGATATGA	60	99.2	0.999	296
RPL13A	F:CCTGGAGGAGAAGAGGAAAGAGA R:TTGAGGACCTCTGTGTATTTGTCAA	60	100.8	0.999	126

### Expression levels of candidate HKGs in epithelial ovarian tissues

**Figure 2:**
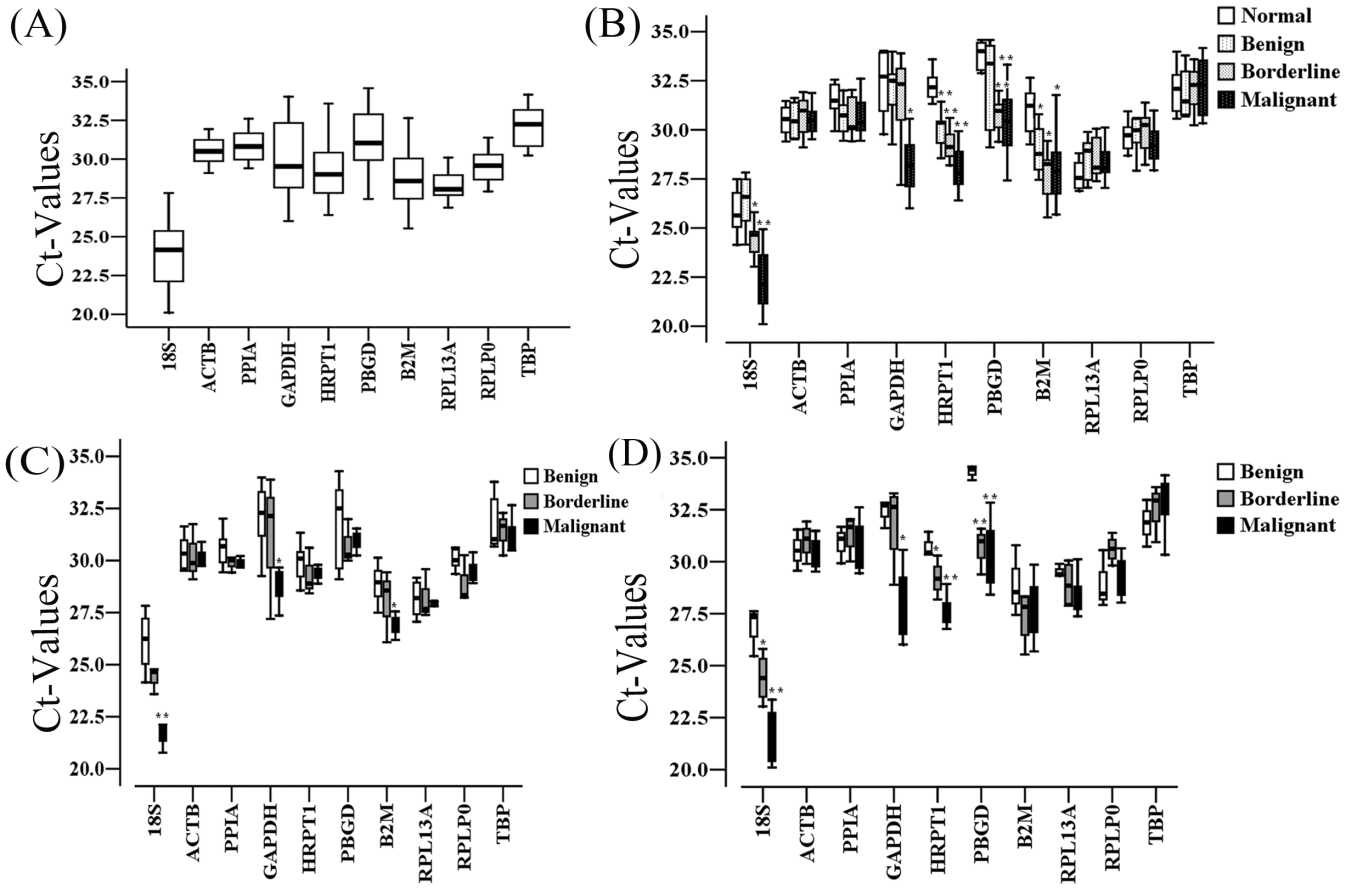
Expression levels of candidate reference genes in ovarian tissues. (A) C_t_ values of the 10 reference genes in fifty ovarian tissues. Boxes reveal the lower and upper quartiles with medians; bars represent the range of the data. (B) Expression changes of the 10 genes in ovarian tissues with four different groups were investigated by one-way ANOVA and the Kruskal-Wallis test. *P<0.05 vs. normal tissues, **P<0.01 vs. normal tissues. (C–D) Expression differences of the 10 genes in mucinous (C) and serous (D) ovarian tissues in three groups were analyzed. *P<0.05 vs. benign tissues, **P<0.01 vs. benign tissues.

The PUM1 and GUSB genes demonstrated C_t_ values above 35 cycles in most of the 50 samples and were expressed at low levels; therefore, PUM1 and GUSB were excluded from further analyses. The remaining 10 HKGs displayed a wide range of expression. The 18S rRNA gene demonstrated the lowest mean C_t_ value (C_t_  = 24), while the TATA-binding protein gene (TBP) exhibited the highest mean C_t_ value (C_t_ = 32.1) (Fig. 2A). One-way ANOVA and the Kruskal-Wallis test were used to compare expression differences that were caused by disease progression. Five genes (ACTB [p = 0.93], PPIA [p = 0.21], RPL13A [p = 0.14], RPLP0 [p = 0.26], and TBP [p = 0.89]) were expressed equivalently in all the 50 specimens from normal, benign, borderline, and malignant tissues; whereas, the expression of the other five HKGs (GAPDH, 18S rRNA, PBGD, HRPT1, and B2M) varied (P<0.05, Fig. 2B). Additionally, the expression levels of B2M, ACTB, PPIA, RPL13A, RPLP0, and TBP were not correlated with patient age (correlation coefficient  = -0.195–0.133; P = 0.174–0.809).

Furthermore, the expression of the 10 HKGs was slightly different between the mucinous and serous neoplasms. Apart from ACTB, PPIA, RPL13A, RPLP0, and TBP, HRPT1 (P = 0.57) and PBGD (P = 0.70) demonstrated relatively equivalent expression in the 13 mucinous ovarian tumors (benign tissues, n = 7; borderline tissues, n = 3; malignant tissues, n = 3, Fig. 2C); whereas, the expression of B2M (P = 0.29) was not significantly different in the 19 serous neoplasms (benign, n = 3; borderline, n = 4; malignant, n = 12, Fig. 2D).

### Determination of HKG expression stability

**Figure 3:**
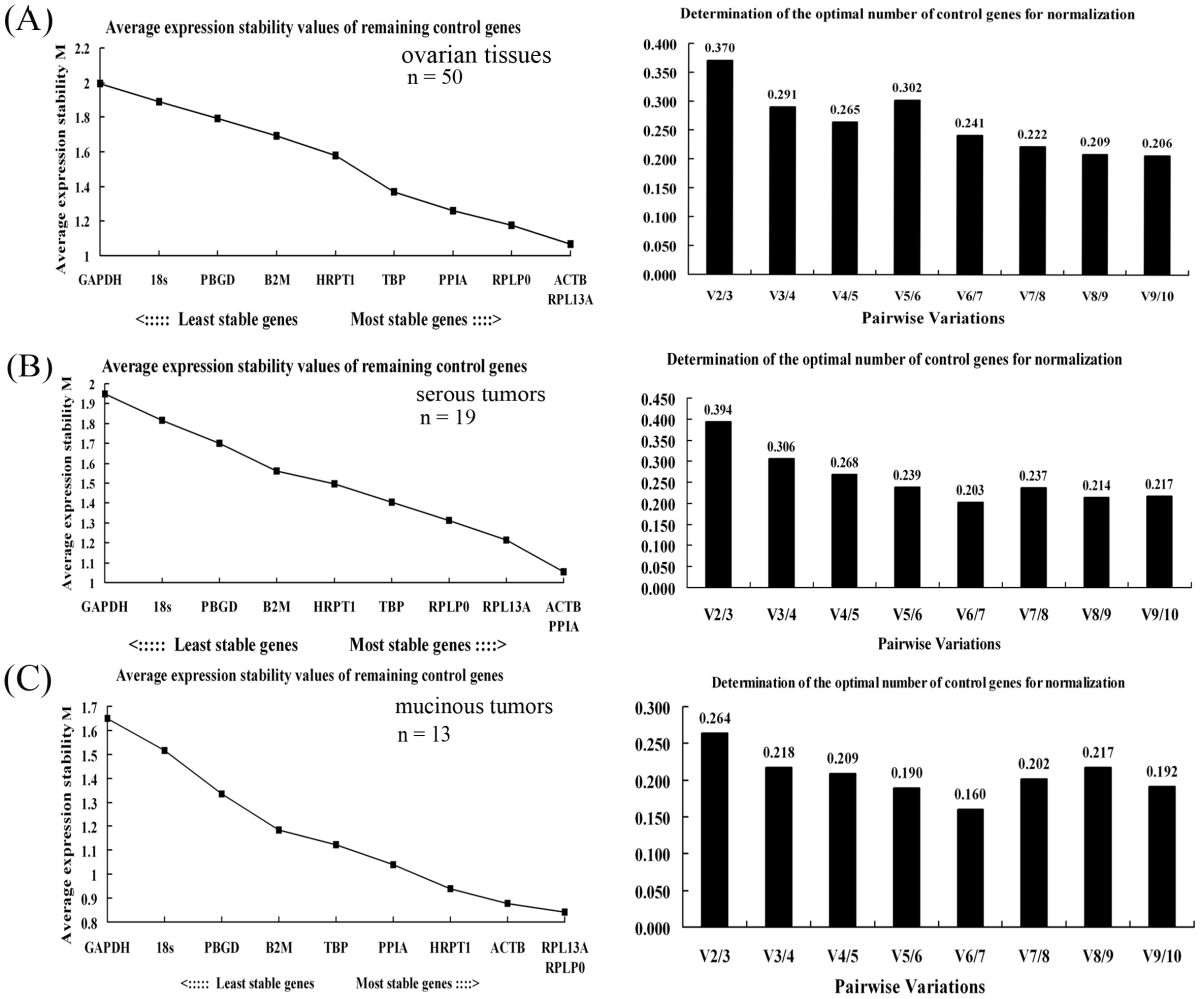
Determination of the expression stability (*M*) of the 10 candidate genes using the geNorm applet. (A) Left, the least stable gene was excluded stepwise by calculating *M* values across 50 ovarian tissues. The average expression stability values and the associated order from least to most stable expression are presented on the y-axis and x-axis, respectively. Right, the determination of the optimal number of internal control genes for normalization depending on the pairwise variation (V) analyses. (B–C) Selection of the most suitable reference genes for normalization in serous (B) and mucinous (C) ovarian tissues using the geNorm program.

The C_t_ values of all 10 candidate reference genes were transformed to relative quantities using the delta-C_t_ method and subjected to two application tools, geNorm and NormFinder. GeNorm calculates the stability of genes (*M*) by pairwise comparisons and then ranks these genes according to their *M* values. With *M* values below the geNorm default threshold of 1.5, TBP, PPIA, RPLP0, ACTB, and RPL13A exhibited high expression stability (Fig. 3A and Table 3). ACTB and RPL13A were identified as the two most stable genes; whereas, GAPDH was the least stable one. Additionally, the geNorm software recommended the combination of four genes (PPIA, RPLP0, ACTB, and RPL13A), which yielded a V4/5 value of 0.265 (Fig. 3A), as a much more reliable normalization strategy.

**Table 3:** Candidate reference genes were ranked according to their expression stability values calculated by geNorm and NormFinder programs.

geNorm		NormFinder	
Gene	Stability value	Gene	Stability value
RPL13A	1.066	PPIA	0.592
ACTB	1.066	RPLP0	0.592
RPLP0	1.176	ACTB	0.660
PPIA	1.259	HRPT1	0.706
TBP	1.367	B2M	0.748
HRPT1	1.580	TBP	0.751
B2M	1.693	PBGD	0.758
PBGD	1.794	RPL13A	0.772
18S	1.889	18S	0.821
GAPDH	1.994	GAPDH	0.993

The NormFinder program analysis revealed varied *M* values ranging from 0.592 to 0.993 (Table 3). GAPDH was still determined to be the least stable gene in our analysis. Whereas PPIA and RPLP0 were identified as the most stable genes, followed by ACTB, HRPT1, and B2M, which were different from the geNorm results. However, HRPT1 (*M* = 0.706) and B2M (*M* = 0.748) should be excluded because each gene was expressed with statistically significant differences in the 50 ovarian tissues. As an alternative to HRPT1 and B2M, the NormFinder results determined that TBP (*M* = 0.751) should be used as a reference gene.

Additionally, the stability of the 10 candidate genes in mucinous and serous neoplasms was also evaluated by these two programs. ACTB, PPIA, RPL13A, RPLP0, TBP, and B2M achieved high expression stability (Fig. 3B, Table 4), which suggested that they were adequate for normalizing gene expression data among ovarian serous tumors. With *M* values less than 1.5, HRPT1, RPL13A, ACTB, TBP, PBGD, and RPLP0 were recommended as a dependable normalizing combination for ovarian mucinous tumors (Fig. 3C, Table 5).

**Table 4:** Stability values of candidate genes in serous tissue samples.

geNorm		NormFinder	
Gene	Stability value	Gene	Stability value
PPIA	1.055	RPL13A	0.504
ACTB	1.055	HRPT1	0.602
RPL13A	1.214	ACTB	0.703
RPLP0	1.313	PPIA	0.784
TBP	1.405	B2M	0.860
HRPT1	1.498	RPLP0	0.983
B2M	1.560	TBP	1.051
PBGD	1.701	PBGD	1.052
18S	1.815	18S	1.158
GAPDH	1.948	GAPDH	1.222

**Table 5:** Stability values of candidate genes in mucinous tissue samples.

geNorm		NormFinder	
Gene	Stability value	Gene	Stability value
RPL13A	0.842	HRPT1	0.366
RPLP0	0.842	RPL13A	0.427
ACTB	0.878	ACTB	0.443
HRPT1	0.940	TBP	0.467
PPIA	1.040	PBGD	0.476
TBP	1.122	RPLP0	0.478
B2M	1.184	B2M	0.482
PBGD	1.335	PPIA	0.576
18S	1.516	18S	0.952
GAPDH	1.649	GAPDH	0.956

## Discussion

Although the combination of LMD and qPCR analysis has been used in ovarian cancers for biomarker and therapeutic target identification [20], [21], for the first time this combination has been employed to HKG selection for ovarian sample research. We confirmed that epithelial cells only occupied a small percentage of the normal ovary, which demonstrated the importance of LMD. From the 12 candidate reference genes we studied, the combination use of ACTB, PPIA, RPLP0, and TBP was identified as a relatively superior normalization strategy, while GAPDH was insufficient for accurate normalization when considering its overexpression in ovarian cancer. These results contribute to reliable normalized qPCR gene expression data in epithelial ovarian cancer.

In this study, we collected approximately 5000 cells from each specimen using an LMD system for RNA extraction and subsequent qPCR. To minimize RNA degradation, the LMD operation must be performed as quickly as possible [6]. Additionally, the use of staining methods that might influence tissue morphology and RNA quality was determined carefully. Cresyl violet, a hydrophilic stain that binds to negatively charged nucleic acids, is recommended as an adequate stain for the identification of target cells and RNA preservation using LMD for qPCR [6], [7]. Furthermore, the RNeasy FFPE kit, which optimizes RNA isolation from microdissected FFPE tissue sections, was also used in this study. Briefly, the comprehensive use of these reagents allowed us to preserve tissue morphology, maximize RNA yield, and protect RNA integrity.

To evaluate candidate gene stability, we employed two statistical models, geNorm and NormFinder [16], [19]. GeNorm identified ACTB, RPL13A, RPLP0, and PPIA as the most stably expressed reference combination in the 50 ovarian samples, while NormFinder highlighted PPIA, RPLP0, ACTB, and HRPT1, followed by B2M and TBP. Given that HRPT1 and B2M were expressed differently in the 50 specimens, which suggested that these two genes were not eligible reference genes, the combination of ACTB, RPLP0, PPIA, and TBP was recommended for use in epithelial ovarian cancer studies. Additionally, to determine the optimal number of reference genes required for normalization, geNorm defines a pairwise variation of 0.15 as the cutoff value, under which the inclusion of an additional gene is unnecessary. However, as Vandesompele *et al.* reported, the proposed 0.15 value must not be considered in a strict sense but rather as a theory guidance [16]. In the present study, the V4/5 value was 0.265, which increased when a gene was included or excluded (V3/4 = 0.29, V5/6 = 0.30). Therefore, the combination of four genes was a preferred choice for normalizing qPCR data in epithelial ovarian cancer. The selection of reference genes for mucinous and serous ovarian cancer also encountered this situation, and the same comprehensive choice was made.

Stability orders of the 10 candidate reference genes determined by NormFinder and geNorm were not completely consistent, which could be explained by the various principles used by each of them. The principle of geNorm is that the expression ratio of two ideal candidate reference genes is identical in all specimens, in spite of disease degree or experimental condition. The geNorm software is suitable for the identification of a combination of at least two genes rather than a single gene. The variation in the expression ratio of two internal control genes reveals that one or both genes is (are) not equivalently expressed, which results in increased variation in the ratio corresponding to decreased expression stability [16]. NormFinder orders the set of HKGs according to their expression stability in a given experimental design and given sample set. It estimates the expression variation of both the intra- and inter-group and provides a stability value for each candidate gene. Compared to geNorm, this applet is less affected by HKG co-regulation. Therefore, in studies with identified inconsistent results, the NormFinder program is preferred to the other programs [19]. Accordingly, in this study, TBP should be superior to RPL13A and, therefore, added into the stable combination for normalization in epithelial ovarian cancer studies.

Additionally, some similar results were attained based on the two applets we used. PPIA, ACTB, and RPLP0 were identified as the most stable genes, and GAPDH and 18S rRNA were considered to be the least stable ones in epithelial ovarian tissues. However, in previous studies [17], [18], ACTB was considered as an unstable normalizer, and 18S rRNA was highlighted as a stable gene which were contrary to our results. This inconsistency is likely due to the differences in the starting materials (whole tissues vs. LMD materials) and in the composition of the samples. In addition, PPIA and the ribosomal protein gene RPLP0 were identified as the most stable reference genes in ovarian tissues by Li *et al.* and Fu *et al.*, respectively. Furthermore, in accordance with our results, Li *et al.* reported that GAPDH was significantly increased in malignant ovarian tissues. The altered expression of GAPDH was also found in other types of neoplasia, such as human glioma, atopic bronchial epithelial cells, and squamous cervical cancer [22]–[24].

In conclusion, for an accurate gene expression assay in ovarian tumor tissues, homogeneous tissues as starting materials and normalization based on multiple validated reference genes, such as PPIA, RPLP0, ACTB, and TBP, are recommended.
